# Simultaneous gastric cancer and breast cancer metastases to the stomach with lymph node collision tumor: a case report

**DOI:** 10.1186/s12876-021-01823-4

**Published:** 2021-05-25

**Authors:** Takeshi Okamoto, Hidekazu Suzuki, Katsuyuki Fukuda

**Affiliations:** 1 Department of Gastroenterology, St. Luke’s International Hospital, 9-1 Akashicho, Chuo-ku, Tokyo, 104-8560 Japan; 2 Department of Gastroenterology and Hepatology, Tokai University School of Medicine, 143 Shimokasuya, Isehara-shi, Kanagawa, 259-1143 Japan

**Keywords:** Gastric cancer, Breast cancer, Collision tumor, Double cancer, Poorly differentiated cancer, Case report

## Abstract

**Background:**

While double primary cancers are common in breast cancer patients, co-existence of primary gastric cancer and gastric metastases of breast cancer is exceedingly rare.

**Case presentation:**

A 51-year-old woman receiving chemotherapy for breast cancer presented with melena and presyncope. A circumferential thickening of the pylorus and small submucosal tumor-like lesions in the gastric fundus and corpus were confirmed on endoscopy. Immunohistochemistry of biopsies revealed that the former was composed of poorly differentiated gastric cancer cells, while the latter were breast cancer metastases. Distal gastrectomy was performed. Pathological evaluation of the resected specimen revealed gastric adenocarcinoma in the pyloric lesion and breast countless cancer metastases throughout the remainder of the stomach, with positive margins. One lymph node had evidence of both stomach cancer and breast cancer metastases, forming a collision tumor. Despite a successful surgery, the patient died 6 months later due to progression of breast cancer.

**Conclusion:**

We report a case of synchronous primary gastric adenocarcinoma and gastric metastases of breast cancer. Inter-disciplinary collaboration is crucial in determining the optimal treatment in double cancers.

## Background

More than 2 million new cases of breast cancer are diagnosed every year. Approximately 6% are metastatic at diagnosis in developed countries, while 20–30% eventually develop metastatic breast cancer [1]. Most common sites of metastases are bone, lung, liver and brain. Gastric metastases are rare, found in up to 0.3% of retrospective studies [2]. A majority result from invasive lobular breast carcinoma and present as secondary linitis plastica, with a characteristic “leather bottle” appearance with stiffened and thickened gastric walls similar to those observed in some gastric signet-ring cell carcinomas [3].

Gastric cancer and breast cancer can occur in the same patient, particularly in women with *CDH1* and *BRCA2* mutations [4, 5]. It can be difficult to differentiate gastric cancer from metastatic breast cancer on clinical, endoscopic, or pathological grounds, although diagnostic accuracy is improving with new immunohistochemical markers [2]. We report a case of synchronous occurrence of primary gastric adenocarcinoma and multiple gastric metastases from breast cancer, both proven by biopsy during the same endoscopic session. Collision of metastases of the two cancers were also confirmed in a resected lymph node.

## Case presentation

A 51-year-old woman presented with melena and presyncope. She had noticed that her stools were black 5 days earlier, with gradual onset of malaise. She felt light-headed the night before and noted dark blood in her stools 1 h before her presentation to the emergency room. She denied any abdominal pain or vomiting.

She had a history of invasive ductal carcinoma (estrogen receptor (ER)-positive, progesterone receptor (PgR)-positive, human epidermal growth factor receptor 2-negative, Ki-67 index: 10.6%) of the left breast measuring over 5 cm, with multiple bone metastases to the spine, sternum, and left ileum, diagnosed 3 years prior. Treatment with leuprorelin acetate, tamoxifen, and denosumab was started. Leuprorelin acetate was replaced with letrozole after 18 months of stable disease, when right pleural effusion and carcinomatous lymphangiosis were noted. Disease progression was again observed 12 months later, leading to commencement of chemotherapy with paclitaxel. While partial response was initially achieved, a splenic lesion suspicious of metastasis had recently appeared. No metastases to lymph nodes, lung, liver, or the contralateral breast were noted.

Other medical history was only remarkable for surgical resection of a benign tumor of the left breast 10 years ago, which was believed to be unrelated to her breast cancer. She had no history of alcohol use, smoking. She also denied recent ingestion of raw foods, overseas travel, or sick contacts. Family history was unremarkable, with no history of cancer in any blood relatives. Screening esophagogastroduodenoscopy (EGD) conducted in the previous year was completely normal, with no signs of atrophic gastritis, ulcers, or tumors.

**Fig. 1:**
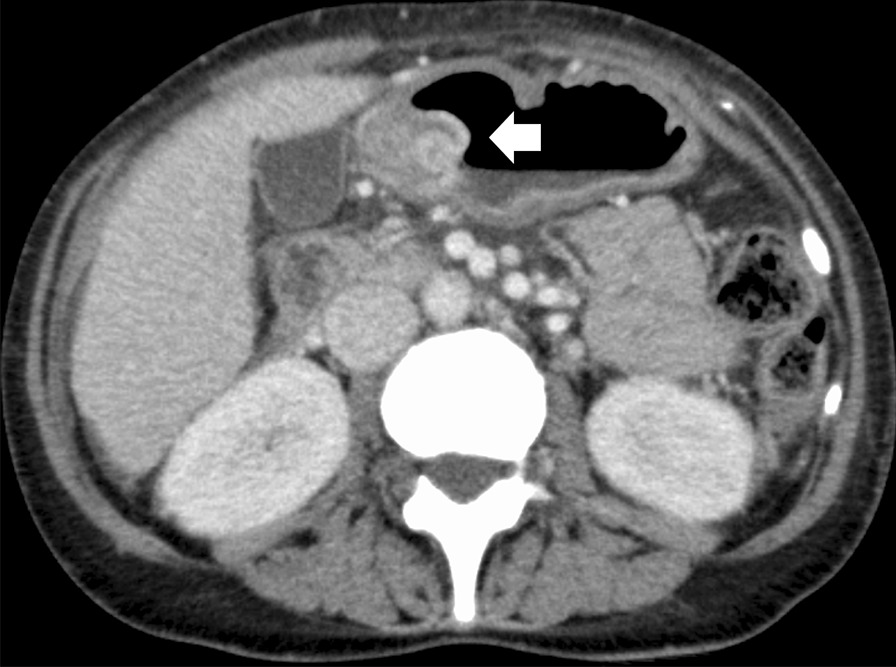
Computed tomography with contrast revealed non-specific thickening of the pylorus (arrow) with no clear signs of gastrointestinal bleeding

Hypotension, tachycardia, and severe anemia (hemoglobin level of 4.6 g/dL) were noted upon presentation. Physical examination of the abdomen was unremarkable. Carcinoembryonic antigen (CEA) had increased from 4.3 to 7.9 ng/mL (reference range: 0–5 ng/mL) and carbohydrate hydrogen 15–3 had increased from 142 to 174 U/mL (reference range: 0–30 U/mL) over a span of 2 months. Computed tomography (CT) with contrast revealed no significant interval change, although non-specific thickening of the pylorus was noted (Fig. 1). There were no clear signs of gastrointestinal bleeding or enlarged lymph nodes.

**Fig. 2:**
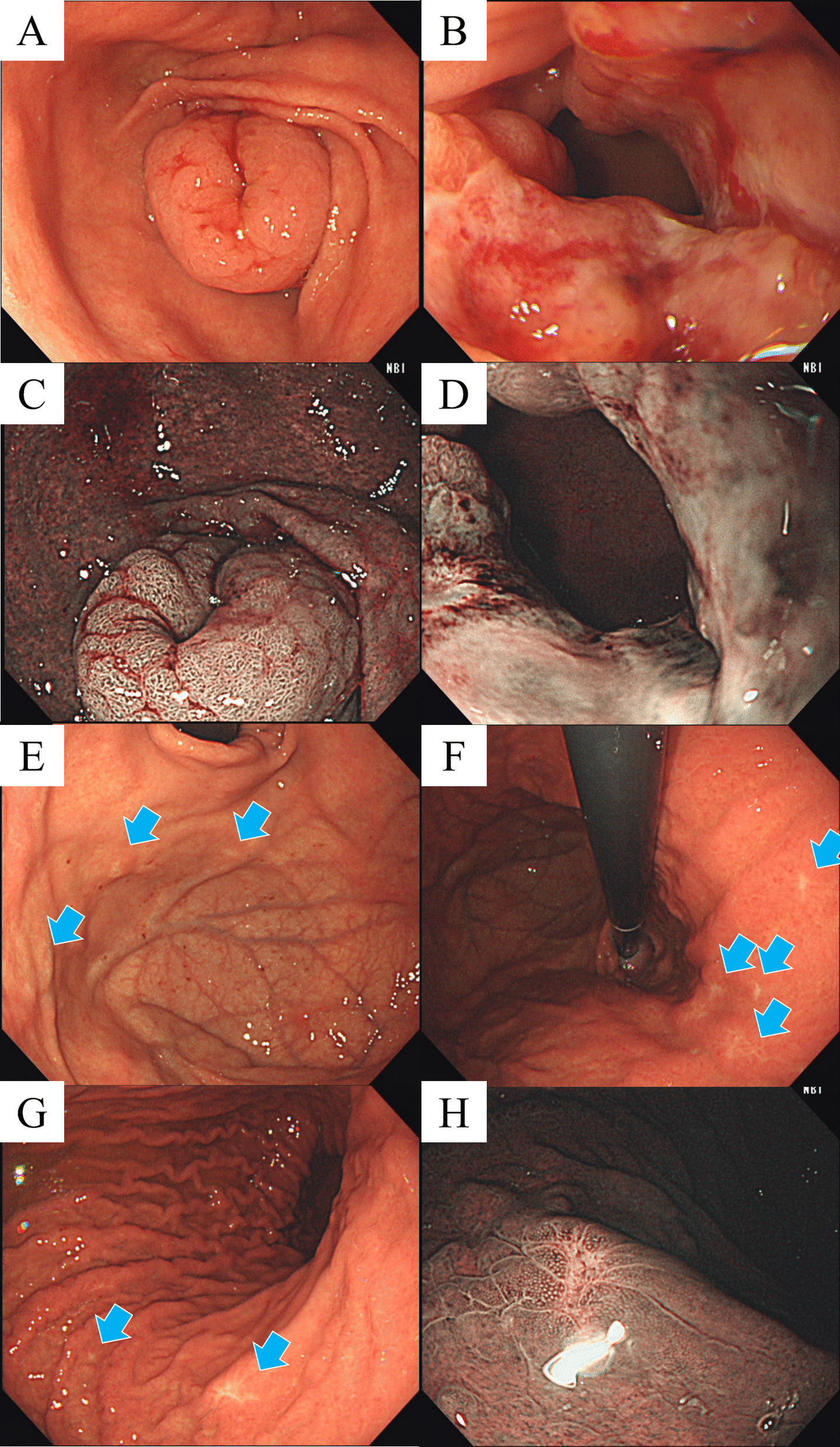
Esophagogastroduodenoscopy. (**A**, **B**) Focal, circumferential thickening of the pylorus with moderate stenosis and ulceration. (**C**, **D**) No mucosal or vascular irregularities suggestive of malignancy were readily apparent on narrow-band imaging (NBI). (**E**–**G**) Numerous blanched, slightly depressed or nodular lesions were observed throughout the gastric fundus and corpus (arrows). (**H**) NBI revealed erosions with slight vascular irregularities surrounded by gastric mucosa with regular but enlarged pits

Emergent EGD revealed focal, circumferential thickening of the pylorus. Slight ulceration with a visible vessel in the six o’clock position of the pyloric ring was noted. Bleeding was observed on contact with the endoscope, and hemostasis was achieved with hemoclips. Follow-up EGD after discharge revealed enlargement of the focal, circumferential thickening of the pylorus (Fig. 2A). Slight ulceration was observed, but no mucosal or vascular irregularities suggestive of malignancy were readily apparent on narrow-band imaging (NBI) (Fig. 2B–D). The pylorus was stenotic, barely allowing the endoscope to pass through. Numerous blanched, slightly depressed or nodular lesions were also observed throughout the gastric fundus and corpus (Fig. 2E–G). NBI revealed erosions with slight vascular irregularities surrounded by gastric mucosa with regular but enlarged pits (Fig. 2H).

**Fig. 3:**
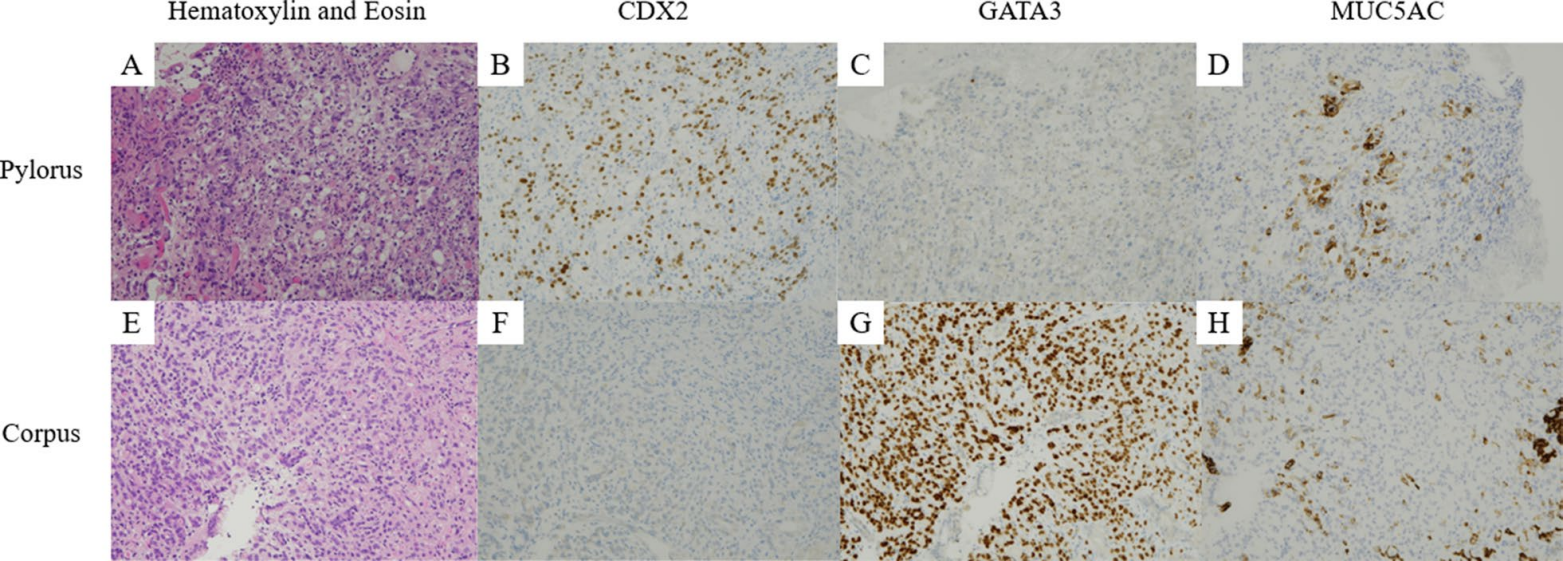
Pathology of endoscopic biopsies. (**A**) Poorly differentiated adenocarcinoma was confirmed on biopsy of the thickened pylorus. (**B**–**D**) The tumor was diagnosed as primary gastric adenocarcinoma based on immunohistochemistry: CDX2-positive, GATA3-negative, and MUC5AC-positive. (**E**) Poorly differentiated adenocarcinoma was also confirmed on biopsies of the depressed lesions in the gastric corpus. (**F**–**H**) The tumor was diagnosed as metastatic breast cancer based on immunohistochemistry: CDX2-ngeative, GATA3-positive, and MUC5AC-negative (the brown areas in the MUC5AC stain are artifacts; the cytoplasm is stained instead of the nucleus)

Pathological evaluation of the biopsies surprisingly revealed two different types of adenocarcinoma (Fig. 3). Both displayed small clusters of atypical cells with increased chromatin condensation consistent with poorly differentiated adenocarcinoma. On immunohistochemistry, only biopsies from the pylorus was positive for cytokeratin 20, CDX2, and MUC5AC. The biopsies from the corpus were immunohistochemically similar to the breast biopsy, staining positive for ER and PgR as well as gross cystic disease fluid protein-15 (GCFDF15), GATA binding protein 3 (GATA3), and mammaglobin. The patient was diagnosed with simultaneous primary gastric adenocarcinoma and gastric metastases from breast cancer.

Surgery to resect only the primary gastric cancer was planned, as the interdisciplinary cancer board determined that gastric cancer would have a larger prognostic impact than breast cancer. Laparoscopy-assisted distal gastrectomy with Billroth-II anastomosis was performed. Small lumps were observed on the serosal side of the stomach, but no clear signs of peritoneal metastases were noted. Distal gastrectomy was completed with some difficulty, due to severe fibrosis surrounding the pyloric tumor.

**Fig. 4:**
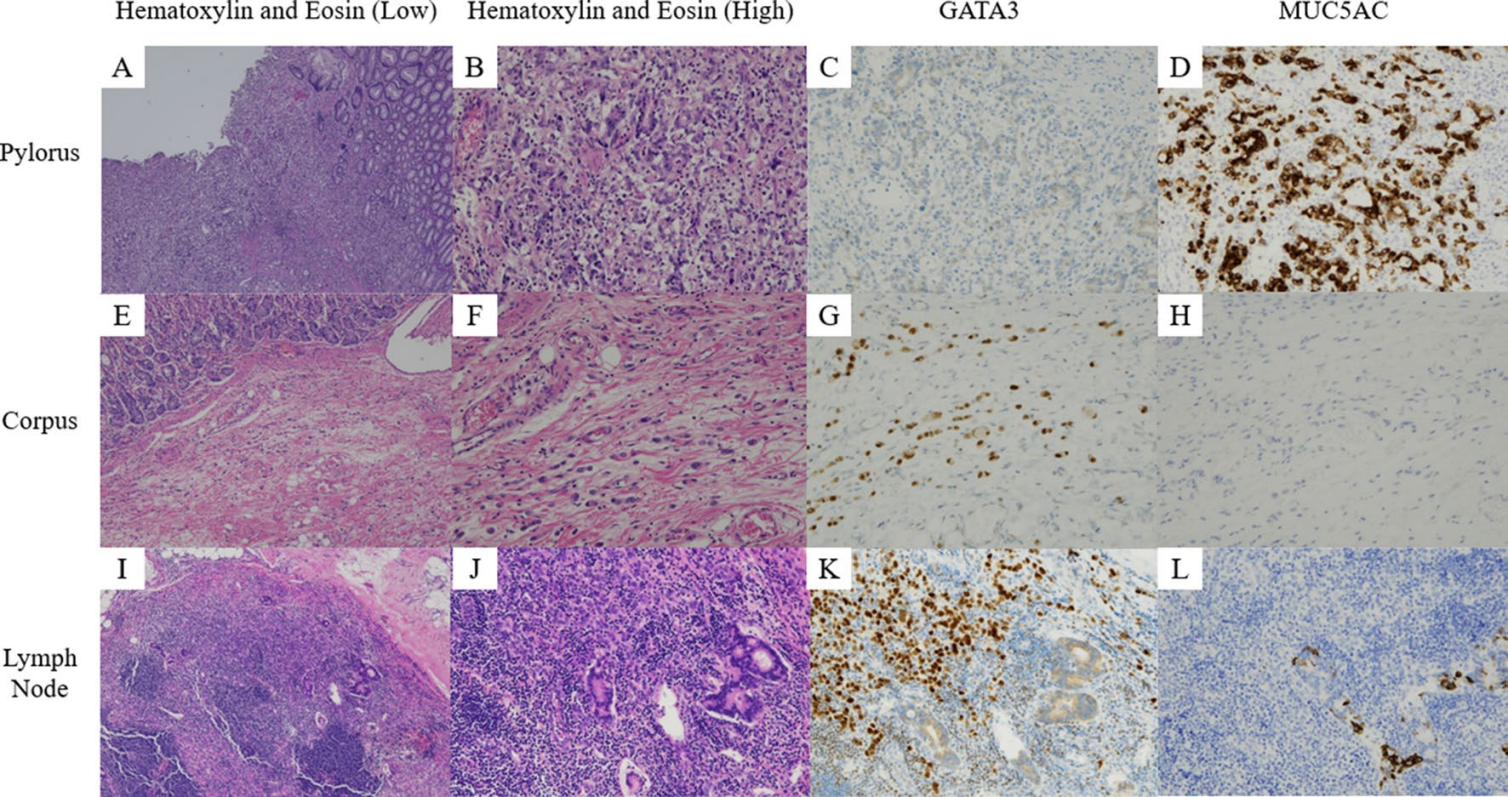
Pathology of the surgical specimen. (**A**–**D**) The pyloric mass was GATA3-negative and MUC5AC-positive, consistent with gastric cancer. (**E**–**H**) The numerous nodules in the corpus was GATA3-positive and MUC5AC-negative, consistent with metastatic breast cancer. (**I**–**L**) A lymph node was partially GATA3-positive/MUC5AC-negative and partially GATA3-negative/MUC5AC-positive, suggesting a collision tumor of metastases from both gastric cancer and breast cancer

Macroscopic examination of the surgical specimen revealed a 35 × 20 × 15 mm lesion in the antrum, with small nodules throughout the remainder of the resected specimen. Hematoxylin and eosin stains of the antral mass revealed poorly differentiated malignant cells reaching the subserosa. All other areas were also occupied by diffuse proliferation of poorly differentiated adenocarcinoma cells, mainly located in the submucosal to subserosal layers but with sparse invasion of the mucosa as well as the serosa. There was a sharp contrast in immunohistochemical stains of the mass and nodules, which were consistent with the preoperative biopsies of the pylorus and corpus, respectively. While surgical margins for the primary gastric adenocarcinoma were negative, both oral and anal margins were positive for breast cancer cells. All resected lymph nodes contained breast cancer metastases. Interestingly, a collision tumor between gastric cancer metastasis and breast cancer metastasis was confirmed in one of the lymph nodes (Fig. 4). The entire specimen was negative for *Helicobacter pylori* infection.

The post-operative course was uneventful. The patient’s breast cancer continued to progress rapidly despite chemotherapy with adriamycin and cyclophosphamide, followed by eribulin mesylate. She opted for palliative care fourth months later and died soon thereafter. Due to the unfavorable clinical course, genetic testing could not be proposed.

## Discussion and conclusion

In contrast to composite tumors which are single tumors with two different cell lines, collision tumors are characterized by the co-existence of two tumors of separate origins in the same specimen, often mixing together with no clear boundaries. Over 50 cases of synchronous gastric adenocarcinoma and lymphoma have been reported in the literature, of which at least five were collision tumors [6, 7]. There are also reports of gastric collision tumors between adenocarcinoma and a benign tumor or between two benign tumors. Lymph node collisions also frequently involve lymphomas, with sparse reports of collision metastases between two solid cancers, including breast cancer [8, 9].

One institution found metachronous double primary cancer in 4.1% of breast cancer patients, of which 14.8% were gastric cancers [10]. The same institution reported metachronous double primary cancer in 3.7% of gastric cancer patients, of which 5.1% were breast cancers [11]. The incidence of stomach cancer increased significantly after treatment for breast cancer (relative risk: 2.61, confidence interval, 1.68–4.06), while the converse did not hold true [10, 11]. While a combination of genetic disposition, common environmental factors, and treatment-related carcinogenesis may be associated with this trend, further research is warranted.

There is only one report of gastric and breast cancers arising in the same organ [12]. The case is similar to ours in that the breast cancer was invasive ductal carcinoma (whereas gastric metastases usually result from invasive lobular carcinoma), the gastric cancer presented with bleeding (whereas gastric metastases are generally asymptomatic or cause obstructive symptoms due to secondary linitis plastica), collision tumors were observed in both the stomach and lymph nodes, and prognosis was limited despite surgery. To the extent of our search, our case is the first to diagnose primary and metastatic solid tumors in the same organ during the same endoscopic session.

Both cancers in our case were poorly differentiated adenocarcinomas which exhibited a primarily subepithelial growth pattern with minimal mucosal changes. While malignant cells were obtained from both types via endoscopic biopsies, there would have been additional diagnostic hurdles if the cancers were completely subepithelial in nature. Immunohistochemistry provided clear evidence of two different cancers in endoscopic biopsies from the same session, the resected stomach, and a resected lymph node (Table 1). CD20, CDX2 and MUC5AC were uniquely associated with the gastric cancer while GCFDF15, GATA3, ER, and PgR were uniquely associated with metastatic breast cancer in our patient. As this is not always the case, hepatocyte nuclear factor 4A immunohistochemistry may also aid in distinguishing primary gastric cancer and breast metastases [2].

**Table 1:** Immunohistochemical properties of malignant lesions

	Breast	Mass in pylorus		Nodules in corpus		Lymph node
	Fine-needle Biopsy	Gastric Biopsy	Surgical Specimen	Gastric Biopsy	Surgical Specimen	Surgical Specimen
CK7		+	+	+	+	
CK20		Focal+	Focal+	–	–	
CDX2		+	+	−	−	
E-cadherin			+		+	
GCDFP15		−	−	+	+	
GATA3		−	−	+	+	Partial
Mammaglobin		−	−	+	+	
MUC1		−	−	+	+	
MUC2		−	−	−	−	
MUC5AC		+	+	−	−	Partial
MUC6		+	+	+	+	
ER	+	−	−	+	+	
PgR	+	−	−	+	+	
HER2	− (score 1)	− (score 0)	−	− (score 1)	+	

*CK* cytokeratin, *ER* estrogen receptor, *GATA3* GATA binding protein 3, *GCDFP15* gross cystic disease fluid protein 15, *HER2* human epidermal growth factor receptor 2, *PgR* progesterone receptor

In conclusion, we report a case of synchronous primary gastric adenocarcinoma and gastric metastases of breast cancer. Inter-disciplinary collaboration is crucial in determining the optimal treatment in double cancers. A high index of suspicion is required to identify metastatic lesions which can mainly invade subepithelial layers of the stomach.

## Data Availability

Not applicable.

## Abbreviations

CEA: Carcinoembryonic antigen
CT: Computed tomography
EGD: Esophagogastroduodenoscopy
ER: Estrogen receptor
GATA3: GATA binding protein 3
GCFDF15: Gross cystic disease fluid protein-15
NBI: Narrow-band imaging
PgR: Progesterone receptor
